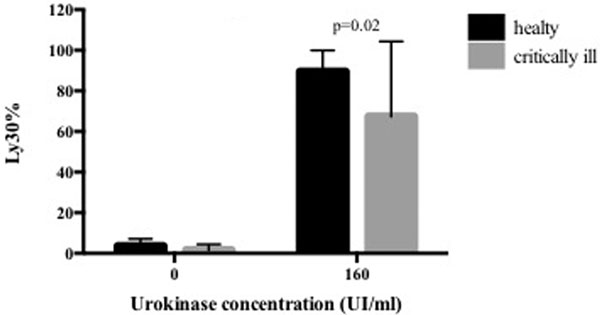# Urokinase-induced fibrinolysis in thromboelastography (UKIF-TEG) to assess fibrinolysis in critically ill patients

**DOI:** 10.1186/cc12295

**Published:** 2013-03-19

**Authors:** L Zacchetti, M Panigada, M Cressoni, T Marchesi, L Gattinoni

**Affiliations:** 1 Fondazione IRCCS Ca' Granda Ospedale Policlinico Milano, Milan, Italy

## Introduction

Coagulopathy, particularly a trend toward hypercoagulability and hypofibrinolysis, is common in critically ill patients and correlates with worse outcome. Available laboratory coagulation tests to assess fibrinolysis are expensive and time demanding. We investigated whether a modified thromboelastography with the plasminogen activator urokinase (UKIF-TEG) [1] may be able to evaluate fibrinolysis in a population of critically ill patients.

## Methods

UKIF-TEG was performed as follows: first urokinase was added to citrate blood to give final concentrations of 160 UI/ml, then thromboelastography (TEG) analysis was started after kaolin activation and recalcification with calcium chloride. Basal TEG (no addition of urokinase) was also performed. Fibrinolysis was determined by the loss of clot strength after the maximal amplitude (MA), and recorded as Ly30 (percentage lysis at 30 minutes after MA) and as Ly60 (percentage lysis at 60 minutes after MA).

## Results

UKIF-TEG was performed on 17 healthy volunteers and 18 critically ill patients. Ly60 was predicted by Ly30 according to an exponential function, so we used Ly30 as an indicator of clot lysis. Basal TEG showed increased coagulability and a trend toward less fibrinolysis in critically ill patients compared with healthy volunteers (reaction time 8.7 ± 3.4 minutes vs. 12.2 ± 1.9 minutes, *P <*0.001; α-angle 59.7 ± 9.4 vs. 47.2 ± 11.8, *P <*0.01). This reduction of fibrinolysis was more evident at a urokinase concentration of 160 UI/ml (Figure 1).

## Conclusion

UKIF-TEG could be a feasible point-of-care method to evaluate fibrinolysis in critically ill patients.

**Figure 1:**